# Interstitial pneumonitis during rituximab-containing chemotherapy for primary central nervous system lymphomas: a case report and review of literature

**DOI:** 10.1186/s41016-017-0106-3

**Published:** 2018-01-08

**Authors:** Yuchen Wu, Xuefei Sun, Jing Liu, Jun Qian, Xueyan Bai, Yuedan Chen, Yuanbo Liu

**Affiliations:** Department of Hematology, Beijing Tiantan Hospital, Capital Medical University, Beijing, 100050 China

**Keywords:** Central nervous system neoplasm, Lymphoma, Interstitial pneumonitis, Rituximab, R-MAD regimen, Rituximab induced lung disease, Chemotherapy, Immunotherapy

## Abstract

**Background:**

Primary central nervous system lymphoma(PCNSL) is a rare kind of non-Hodgkin lymphoma. Rituximab combined with high-dose methotrexate, cytarabine and dexamethasone (R-MAD regimen) were reported effective for PCNSL patients. Rituximab can cause several side effects, including fever, chills and rigors.

**Case presentation:**

In this case report, we demonstrate rituximab-induced interstitial pneumonitis in a PCNSL patient who has been treated with R-MAD regimen. The patient recovered after treatment and she remains complete remission after following consolidation chemotherapy.

**Conclusions:**

Here is no report of potential fatal complications of Rituximab like interstitial pneumonitis nowadays in PCNSL patients. As Rituximab is widely used, physicians should raise their awareness of this rare complication and detect RTX-ILD in early stage.

## Background

Primary central nervous system lymphoma(PCNSL)are extranodal Non-Hodgkin lymphomas confined to the brain, eyes, leptomeninges, or spinal cord in the absence of systemic lymphoma,and is predominantly (90–95%) of the diffuse large B-cell lymphoma (DLBCL) subtype [1]. Methotrexate containing regiment(high-dose methotrexate(MTX) with cytarabine(Ara-C) and dexamethasone) is presently considered as the first choice for PCNSL patients whose preparing to take chemotherapy. Rituximab(RTX) is a chimeric anti-CD20 monoclonal antibody. It is reported that the complete response rate and overall survival in PCNSL accessed to high-dose methotrexate-contained regiment can be improved with the addition of Rituximab [2]. We previously retrospective analyzed 35 PCNSL patients in hematology department of Beijing Tiantan hospital who received R-MAD regimen and prove its safety and effectiveness [3]. During the infusion of RTX, there can be some common side effect including fever, chills, and rigors. Severe respiratory conditions have never been reported in PCNSL patient before. To raise awareness of this serious complication of RTX containing treatment, we demonstrate rituximab-induced interstitial pneumonitis in a PCNSL patient who has been treated with R-MAD(Rituximab, high-dose methotrexate, cytarabine, and dexamethasone) regimen.

## Case description

**Fig. 1:**
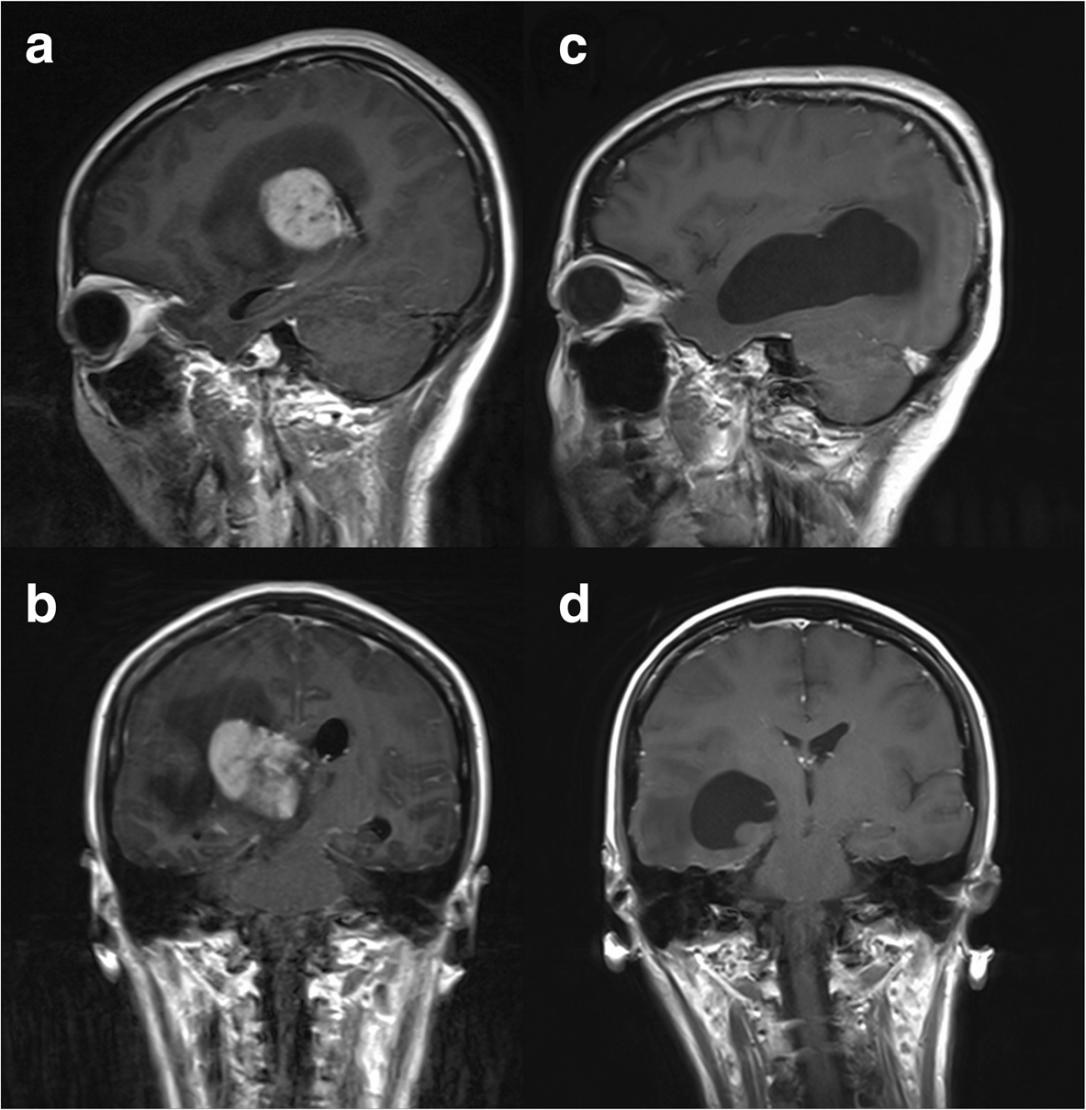
**a**, **b** MRI enhancement revealed: on the right side of the thalamus a block of long T2 long T1 signal can be seen,the boundary is clear, the range is 44 × 40 × 34 mm. Enhanced scanning showed inhomogeneous enhancement. Massive edema can be seen around the lesion. The third ventricle was compressed,bilateral ventricles dilated. Midline shift left slightly. **c**, **d** MRI enhancement after surgery and 5 cycles of R-MAD chemotherapy showed: right side of the occipital skull and soft tissue post operation change. The right lateral ventricles triangle and the temporal angle were irregular enlarged, the ventricular wall was less structured

Four months ago, a 33y female patient complained low fever with acratia, night sweating, and disorder of left limb’s activity. Her brain CT showed right lateral ventricle occupying lesion. Postoperative pathology showed diffuse large B-cell lymphoma, after which she received 5 cycles of R-MAD regimen (Rituximab 600 mg d1, high-dose methotrexate, cytarabine, and dexamethasone) chemotherapy (Fig. 1). Then her left limbs activity fully recovered.

Two weeks after the fifth cycle of R-MAD regimen chemotherapy (Rituximab 600 mg d1, high-dose methotrexate, cytarabine, and dexamethasone), she was admitted to hospital with the symptoms of fever and abdominal pain. The physical examination revealed temperature 38.7 °C; pulse 82/min; respiration 20 times/min; blood pressure 120/80 mmHg; Clear consciousness; mild anemic appearance; no swelling of superficial lymphnode. Her chest was clear to percussion and auscultation, and her heart and abdomen showed no abnormality, also, her lower limbs has no swelling. Blood routine: white cell count(WBC) 4.68*10^9^/L, percentage of neutrophilic granulocyte 75.9%, hemoglobin 119 g/l, platelet count 55*10^9^/L. Stool routine: red blood cell (− /HPF), white blood cell count(− /HPF), occult blood(−), fungi(− /HPF).

**Fig. 2:**
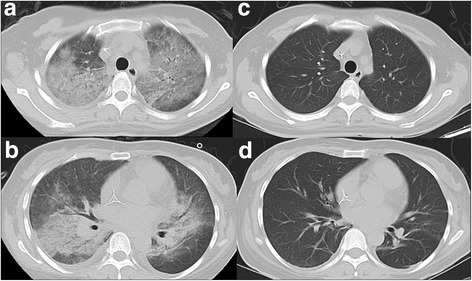
**a**, **b** Helical computed tomographic scanning showing bilateral diffuse ground glass opacities, partialpleura thickening, crescent shaped liquid density of bilateral chest wall. **c**, **d** A repeat CT scan 7 days after glucocorticoid and antibiotic therapy showing almost complete resolution of the interstitial infiltration

Preliminary diagnosis was a gastroenteritis caused by unhygienic food. Cefmetazole were prescribed for her. After using cefmetazole for 1 day, her temperature raised up to 40 °C.Blood routine: white blood cell count 2.08*10^9^/L, neutrophilic granulocyte 1.26*10^9^/L, hemoglobin 101 g/l, platelet count 80*10^9^/L. C-reactive protein 20.05 mg/L. Procalcitonin <0.05 ng/ml. X-ray examination showed intensification of pulmonary grains, bilateral exudative change, suggesting pulmonary infection. So, meropenem was given. Though the patient complained no cough, no expectoration or dyspnea, she had cyanosis and bilateral coarse breath sounds on pulmonary auscultation. Blood gas demonstrated type I respiratory failure (pH 7.44, PaO_2_41mmHg, PaCO_2_34 mmHg, SaO_2_ 78%). Chest CT demonstrated bilateral lung fields with diffuse ground glass opacities, compatible with interstitial pneumonitis (Fig. 2).

Blood routine, procalcitonin, c-reactive protein retesting results suggested severe infectious pneumonia. If the infection of pulmonary was the only existing pathogenesis, the patient should have responded to antibiotics treatment, but turned out not. Thus, rituximab induced lung disease (RTX-ILD) was considered possible. After that high-dose intravenous steroid (80 mg urbason) was given immediately with continuous meropenem treatment. One day after intravenous steroid, her temperature back to normal. Arterial blood gas changes see in Table 1. CT reexamination revealed significant lesion absorption, and normal pulmonary grain after seven-day steroid treatment (Fig. 2). In addition, her chest was clear to percussion and auscultation, and her saturation of blood oxygen was at normal level. After that, the patient received two cycles of pemetrexed and two cycles of EA regimen (Etoposide and cytarabine) as consolidation chemotherapy. There were no signs of recurrence of lymphoma on the following MRI and PET scan of the patient.

**Table 1:** Change of arterial blood gas

	Day 1	Day 2	Day 5
PH	7.44	7.44	7.52
Partial pressure of carbon dioxide(PaO_2_)/mmHg	34	36	38
Partial pressure of oxygen(PO_2_)/mmHg	41	156	81
Saturation of oxygen/%	78	97	97
PO_2_/FiO_2_	195	390	324

## Discussion

Rituximab(RTX) has been proved effectively in treating malignant and autoimmune disease including: lymphoma, leukemia, rheumatoid arthritis, Wegeners granulomatosis [4], and benefit non-Hodgkin’s lymphoma (NHL) and RA patients [5, 6]. Infusion-related symptom complex have been observed during the use of RTX which consists fever, chills and rigors. During clinical trials, 9-15% of the patients showed symptoms of infusion-related reactions, while 30% of patients had respiratory manifestations (cough, bronchospasm, sinusitis and rhinitis). These complications are generally mild and self-limited. Severe side effects such as anaphylactic shock and acute respiratory distress syndrome were rare, fatal in 0.04-0.07%. Interstitial lung disease is one of those potentially fatal complications of RTX therapy, it is reported that the overall incidence of RTX-ILD is less than 0.03% in phase III trials [7]. However, it is reviewed that in post-marketing literature the incidence is much higher, ranging from 3.7 -10% [4].The variance between these two incidences might due to different in target population. Phase III trials shown that RTX-ILD is of more severity in lymphoma than in rheumatological disorders, which may due to the difference of dosage used in treatment [8]. As for hematological disorders, the mean dosage of RTX was 7785 mg comparing to 2000 mg in rheumatological disorders. RTX is widely used for patients with B cell lymphoma and the majority case reports are from non-Hodgkin lymphoma patients. According to the previous research of our department, we found that with the use of RTX, the probability of complete response and overall survival in newly diagnosed PCNSL patients had been improved [1]. And this is the first time that R-ILD has been discovered in PCNSL patients.

The pathogenesis of R-ILD remains unclear. One hypothesis is pathogenic cytokine release. Tumor necrosis factor-α(TNF-α) and interleukins are thought to be the dominating contributing cytokines [9, 10]. Recently there was case reported that Nod-like receptor pyrin domain-containing protein 3(NLRP3)may act as an initiator of inflammation process in lung of R-ILD patient. The discovery of NLRP3 and related further researches could open a new sight into the pathologic mechanism and provide a new target for the treatment of RTX-ILD [11].

Rituximab induced lung disease have higher incidence in male and patient of their fifty to sixty. Mostly occurring 2 weeks after the latest cycle of infusion, the fourth cycle of RTX [12], average dose accumulated to 1500 mg/m^2^. Common complains of RTX-ILD included: dyspnea and cough. Other symptoms like fatigue, rigours, wheeze, hemoptysis, skin rash have also been reported. About 20.7% of the cases is detected radiologically without symptoms. Physical examinations may be unremarkable or without diffuse fine inspiratory crackles. As for typical radiological abnormalities, chest radio graph displaying diffuse bilateral lung infiltrates. In high-resolution or helical thoracic CT, ground-glass opacification(GGO), alveolitis, pulmonary fibrosis, alveolar hemorrhage, pleural effusions and consolidation can also be seen. PET-CT could indicate the activation of neutrophil within the lungs by showing 18-FDG accumulation in hypermetabolic lung nodules. Lung biopsy is not usually performed in RTX-ILD, in our case, the diagnosis was made on clinical and radiological basis. Her respiratory symptoms relieved greatly after steroid treatment thus we didn’t see the further need for invasive investigation. Anyway, pulmonary inflammation is a common feature in reported cases. A recent systematic review of the available studies showed various histological patterns including: organizing pneumonia, interstitial pneumonitis, desquamative interstitial pneumonia, diffuse alveolar damage, and usual interstitial pneumonia [13].

According to Wagner’s review, hypoxemia and saturation of blood oxygen decreasing is uniformly abnormal in RTX-ILD [14]. There’s also research signify fever was the most common presenting symptom [15].

In our case report, the patient was free of respiratory symptoms and no abnormal objective sign in lung except for cyanosis is a crucial clue, suggesting hypoxemia. Following arterial blood gas testing confirmed type I respiratory failure. Pulmonary function tests reveal restrictive pattern on spirometry and significant reduction in carbon monoxide diffusion capacity. Further test could be done to confirm diagnosis and evaluate the efficacy of treatment, like transbronchial lung biopsy, bronchoalveolarlavage. Blood culture, sputum culture, antineutrophil cytoplasmic antibody, procalcitonin, antibodies and DNA testing for acute virus and empirical treatment with antibiotics should be down to rule out bacterial infection.

Once RTX-ILD was considered, RTX treatment should be stopped immediately and provide intravenous steroid. Supporting treatment include oxygen-inspiration and noninvasive mechanical ventilation can be provided contingent on blood oxygen. RTX-ILD patients who do not respond to glucocorticoids is potential fatal. In a 21-day treatment, 5 mg of dexamethasone or 40 mg of methyl-prednisolone were used in the first three consecutive days, followed by prednisone taken orally with slow taper.^14^ Treatment for such severe RTX-ILD is still challenging. Some authors have suggested anti-TNFα therapy in severe cases. The first TNF-α antagonist and cytokine profiling treating was published by Yong-Kang Wu, but the he did not obtain an ideal outcome. Moreover, some clinical trials even indicated that TNF-α antagonists themselves may lead to a worse prognosis [16].

The retreatment of rituximab may probably deteriorate pulmonary condition among the patients who had experience R-ILD [17, 18]. However some other reports presented opposite result. Those patients who has been retaken Rituximab contained regimens showed uneventful outcome [19]. Therefore, the decision should be made carefully. As the probability of curing the disease may be improved while the incident rate of potential pulmonary toxicity may be improved as well [14].

## Conclusion

Rituximab is widely used in non-Hodgkin lymphomas including PCNSL. Rituximab induced lung disease is a rare yet potential fatal complication. Early diagnosis and treatment including intravenous glucocorticoids, oxygen-inspirationare are of great importance. To detect RTX-ILD in early stage, physicians subscribing RTX should be clearly aware of its common and rare complications, once patients applying RTX appear cyanosis or hypoxemia, futher examinations including arterial blood gas test and chest CT should be done as soon as possible. The pathogenesis of RTX-ILD remains unclear, NLRP3 may provide a new target for treatment in the future.

## Abbreviations

Ara-C: Cytarabine
CNS: Central nervous system
EA: Etoposide and cytarabine
GGO: Ground-glass opacification
HPF: High power field
MTX: Methotrexate
NHL: Non-Hodgkin’s lymphoma
NLRP3: Nod-like receptor pyrin domain-containing protein 3
PaCO_2_: Partial pressure of carbon dioxide of artery
PaO_2_: Partial pressure of oxygen of artery
PCNSL: Primary central nervous system lymphoma
RA: Rheumatoid arthritis
R-MAD regimen: Rituximab,high-dose methotrexate, cytarabine and dexamethasone
RTX: Rituximab
RTX-ILD: Rituximab induced lung disease
SaO_2_: Arterial oxygen saturation
TNF-α: Tumor necrosis factor-α
WBC: White blood cells
